# Mutant HTT does not affect glial development but impairs myelination in the early disease stage

**DOI:** 10.3389/fnins.2023.1238306

**Published:** 2023-07-19

**Authors:** Sitong Yang, Jingjing Ma, Han Zhang, Laiqiang Chen, Yuxuan Li, Mingtian Pan, Hongcheng Zhu, Jun Liang, Dajian He, Shihua Li, Xiao-Jiang Li, Xiangyu Guo

**Affiliations:** Guangdong Key Laboratory of Non-human Primate Research, Guangdong-Hongkong-Macau Institute of CNS Regeneration, Jinan University, Guangzhou, China

**Keywords:** Huntington’s disease, glia, development, demyelination, oligodendrocyte

## Abstract

**Introduction:**

Huntington’s disease (HD) is caused by expanded CAG repeats in the huntingtin gene (HTT) and is characterized by late-onset neurodegeneration that primarily affects the striatum. Several studies have shown that mutant HTT can also affect neuronal development, contributing to the late-onset neurodegeneration. However, it is currently unclear whether mutant HTT impairs the development of glial cells, which is important for understanding whether mutant HTT affects glial cells during early brain development.

**Methods:**

Using HD knock-in mice that express full-length mutant HTT with a 140 glutamine repeat at the endogenous level, we analyzed the numbers of astrocytes and oligodendrocytes from postnatal day 1 to 3 months of age via Western blotting and immunocytochemistry. We also performed electron microscopy, RNAseq analysis, and quantitative RT-PCR.

**Results:**

The numbers of astrocytes and oligodendrocytes were not significantly altered in postnatal HD KI mice compared to wild type (WT) mice. Consistently, glial protein expression levels were not significantly different between HD KI and WT mice. However, at 3 months of age, myelin protein expression was reduced in HD KI mice, as evidenced by Western blotting and immunocytochemical results. Electron microscopy revealed a slight but significant reduction in myelin thickness of axons in the HD KI mouse brain at 3 months of age. RNAseq analysis did not show significant reductions in myelin-related genes in postnatal HD KI mice.

**Conclusion:**

These data suggest that cytoplasmic mutant HTT, rather than nuclear mutant HTT, mediates myelination defects in the early stages of the disease without impacting the differentiation and maturation of glial cells.

## 1. Introduction

Huntington’s disease (HD) is caused by an expansion of CAG repeats in the huntingtin gene (HTT), resulting in mutant HTT carrying an expanded polyglutamine repeat (more than 36Q) in its N-terminal region (Ross and Tabrizi, 2011; Ross et al., 2014; Saudou and Humbert, 2016). While mutant HTT causes late-onset neurodegeneration in HD, it has been found to affect neuronal cells during early brain development (Reiner et al., 2003; Godin et al., 2010; McKinstry et al., 2014; Molina-Calavita et al., 2014; Molero et al., 2016; Barnat et al., 2020; Mangin et al., 2020; Capizzi et al., 2022). This raises an important hypothesis that the effect of mutant HTT on the early development of the brain may contribute to late-onset neurodegeneration (Cepeda et al., 2019; van der Plas et al., 2020; Humbert and Barnat, 2022). There is also mounting evidence that mutant HTT can affect glial cells, impairing neuronal function (Shin et al., 2005; Bradford et al., 2009, 2010; Khakh and Sofroniew, 2014; Huang et al., 2015; Diaz-Castro et al., 2019; Lee et al., 2022). However, it remains unknown whether mutant HTT affects the development of glial cells during early brain development.

Addressing this issue is important for understanding the effect of mutant HTT during early brain development, as neuronal maturation and function are critically dependent on glial function in the early development stage (Haydon, 2001; Allen and Lyons, 2018; Liu et al., 2023). For example, axonal formation and function rely on myelination produced by oligodendrocytes (Franklin and Ffrench-Constant, 2017; Kramer-Albers and Werner, 2023), while synaptic function is regulated by astrocytes that can uptake neurotransmitters and regulate their release (Blanco-Suarez et al., 2017; Endo et al., 2022). Furthermore, neurotrophic factors provided by glial cells are essential for maintaining neuronal survival and regulating their differentiation and maturation during the early brain development stage (Park and Poo, 2013; Caldwell et al., 2022; Wang et al., 2022; Xie et al., 2022).

Although previous work has shown that mutant HTT can affect neuronal development (Wiatr et al., 2018; Barnat et al., 2020; Fyfe, 2021; Hickman et al., 2021; Capizzi et al., 2022), it is unclear whether glial dysfunction can contribute to this early defect. In the current study, we used the HD KI mouse model to address this issue. HD KI mice express full-length mutant HTT at the endogenous level, allowing us to investigate the toxic effect of mutant HTT under physiological conditions and during early brain development. We focused on astrocytes and oligodendrocytes, as both come from the same progenitor cells. We assessed the numbers of astrocytes and oligodendrocytes in postnatal HD KI mice and observed no differences in their numbers between HD KI and WT mice. However, we found that mutant HTT can reduce myelin protein expression and myelination at 3 months of age. These findings suggest that mutant HTT does not affect the development of glial cells but can impair myelination at the early disease stage, providing additional insight into the neuropathology of HD.

## 2. Materials and methods

### 2.1. Animals

All animal protocols were approved by the Institutional Animal Care and Use Committee (IACUC) of Jinan University in China (IACUC Approval No. IACUC-20200728-01). Wild-type C57BL/6 mice were purchased from the Guangdong Medical Laboratory Animal Center (license No. SCXK (Yue) 2018–0002) and used as controls. HD KI-140Q mice expressing full-length mutant HTT were obtained from Jackson Lab (stock number: 029928). All mice were housed in the animal facility of the Institute of Central Nerve Regeneration at Jinan University, which had a 12-h light period and a 12-h dark period, with a controlled temperature of 22 ± 2°C and humidity of 50 ± 10%. The animals had *ad libitum* access to mouse diet and sterile water.

### 2.2. Western blotting analysis

For Western blotting analysis, brain tissues were homogenized in RIPA buffer (50 mM Tris, pH 8.0, 150 mM NaCl, 1 mM EDTA pH 8.0, 1 mM EGTA, pH 8.0, 0.1% SDS, 0.5% DOC, and 1% Triton X-100) with 1× protease inhibitor (Sigma, P8340). The tissue lysates were then diluted in 1× SDS sample buffer (62.6 mM Tris–HCl, pH 6.8, 2% SDS, 10% glycerol, and 0.01% bromophenol blue) and sonicated for 10 s after incubation at 100°C for 5 min. The total lysates were resolved in a 4–20% Tris-glycine (Invitrogen) and blotted onto a nitrocellulose membrane. The Western blots were developed using the ECL Prime kit (GE Health Care/Amersham Biosciences).

### 2.3. Immunofluorescence staining

Mice were anesthetized with 5% chloral hydrate and then perfused with 0.9% NaCl, followed by 4% paraformaldehyde (PFA). The brains were subsequently removed and post-fixed in 4% PFA overnight at 4°C. The brains were then transferred to 30% sucrose for 48 h and cut into 20-or 40-μm sections using a cryostat (Leica CM1850) at 20°C. The sections were blocked in 4% donkey serum with 0.2% Triton X-100 and 3% BSA in PBS for 1 h. For immunofluorescent staining, 20-μm sections were incubated with primary antibodies in the same buffer at 4°C overnight. After washing with 1× PBS, the sections were incubated with fluorescent secondary antibodies. Fluorescent images were acquired using a Zeiss microscope (Carl Zeiss Imaging, Axiovert 200 MOT) and either a 40× or 63× lens (LD-Achroplan 40×/0.6 or 63×/0.75) with a digital camera (Hamamatsu, Orca-100) and Openlab software (Improvision).

### 2.4. RNAseq analysis

Total RNA was extracted from the prefrontal cortex (PFC) and striatum of 140Q KI mice and age-matched control animals. Only samples with an RNA integrity number (RIN) over 6.8 were used for cDNA library construction. Sequencing was performed on a single lane of an Illumina HiSeq 4,000 to produce 150 bp paired-end reads. We performed three independent replicates from adjacent areas for each animal. The brain tissues were transported on dry ice to BGI Genomics (Shenzhen, China) for high-throughput sequencing. The RNA-seq sequencing workflow followed the company’s protocol. The sequencing libraries were enriched and constructed with magnet beads containing Oligo (dT) and randomly fragmented using fragmentation buffer. The RNA fragments were then amplified using random hexamers, end-repaired, adenylated, and sequenced using the Illumina platform.

The RNA-seq data were quantified using Salmon software (ver 1.9.0) based on the GRCm38 genome on the high-performance computing platform of Jinan University (Patro et al., 2017). The quantified files were then matrixed with tximport (ver 1.28.0) and estimated through edgeR to explore differentially expressed genes (DEGs) (Robinson et al., 2010). The heatmaps were clustered and plotted using the ComplexHeatmap R package (ver 2.13.1) (Gu et al., 2016). The significant up-regulated and down-regulated genes (*p* < 0.01, |Fold Change| > 1) in the cortex and striatum between WT and HD140Q mice were shown on the volcano plot through the EnhanceVolcano R package (ver 1.14.0). To estimate different pathway activation scores in samples, the neuron or glial cell-associated pathway sets scores were calculated through Gene Set Variation Analysis (GSVA) with the GSVA R package (ver 1.48.1) (Hänzelmann et al., 2013). All of the above analyses were performed using R (ver 4.6.0) and R studio (ver 2022.07.01, Build 554).

### 2.5. Quantitative PCR

For qRT-PCR, total RNA was extracted from the prefrontal cortex and striatum of postnatal mice at 3 months of age. Samples were collected from 140Q KI mice and age-matched control animals. Reverse transcription reactions were performed using the Superscript III First-Strand Synthesis System (Invitrogen, 18,080–051) with 1.5 μg of total RNA. One microliter of cDNA was combined with 10 μL SYBR Select Master Mix (Applied Biosystems, 4,472,908) and 1 μL of each primer in a 20 μL reaction. The reaction was performed in a real-time thermal cycler (Eppendorf, Realplex Mastercycler). The PCR products were analyzed on a 2% agarose gel to ensure that the PCR amplification produced only a single specific band. The sequences of the primers for Ptgds, Acvr2a, Ccn3, Rxrg, Nrp2, Vip1r, Igf2, Lars2, Agt, Lgfbp2, Sema7a, Sparc, Bdnf, Hap1, Sox11 and Actin are listed in Supplementary Figure S1. Actin was used as the internal control. Relative expression levels were calculated using 2^-ΔΔCT, with WT set at 1.

### 2.6. Electron microscopy

The mice were anesthetized with 5% chloral hydrate and perfused with 0.9% NaCl, followed by 4% PFA containing 2.5% glutaraldehyde. After post-fixation, the brain was cut into 50-μm sections using a vibratome (Leica, VT1000). All sections used for electron microscopy were dehydrated in ascending concentrations of ethanol and propylene oxide/Eponate 12 (1:1) and embedded in Eponate 12 (Ted Pella, Redding, CA). Ultrathin sections (60 nm) were cut using a Leica Ultracut S ultramicrotome. Thin sections were counterstained with 5% aqueous uranyl acetate for 5 min, followed by Reynolds lead citrate for 5 min, and examined using a Hitachi (Tokyo, Japan) H-7500 electron microscope equipped with a Gatan Bio-Scan CCD camera. Axon and myelin fiber diameters were measured using ImageJ (NIH). More than 60 axon sections were examined for each genotype.

### 2.7. Statistics

The results are presented as mean ± standard error (SE). Statistical analysis was performed using Prism 8 software (GraphPad Software). When comparing only two experimental groups, Student’s t-test was used to calculate statistical significance. For all other experiments, statistical significance was calculated using one-way ANOVA or two-way ANOVA, followed by Tukey’s multiple-comparisons test. A value of p of less than 0.05 was considered statistically significant.

## 3. Results

### 3.1. Selective reduction of myelin proteins in HD KI mice

**Figure 1:**
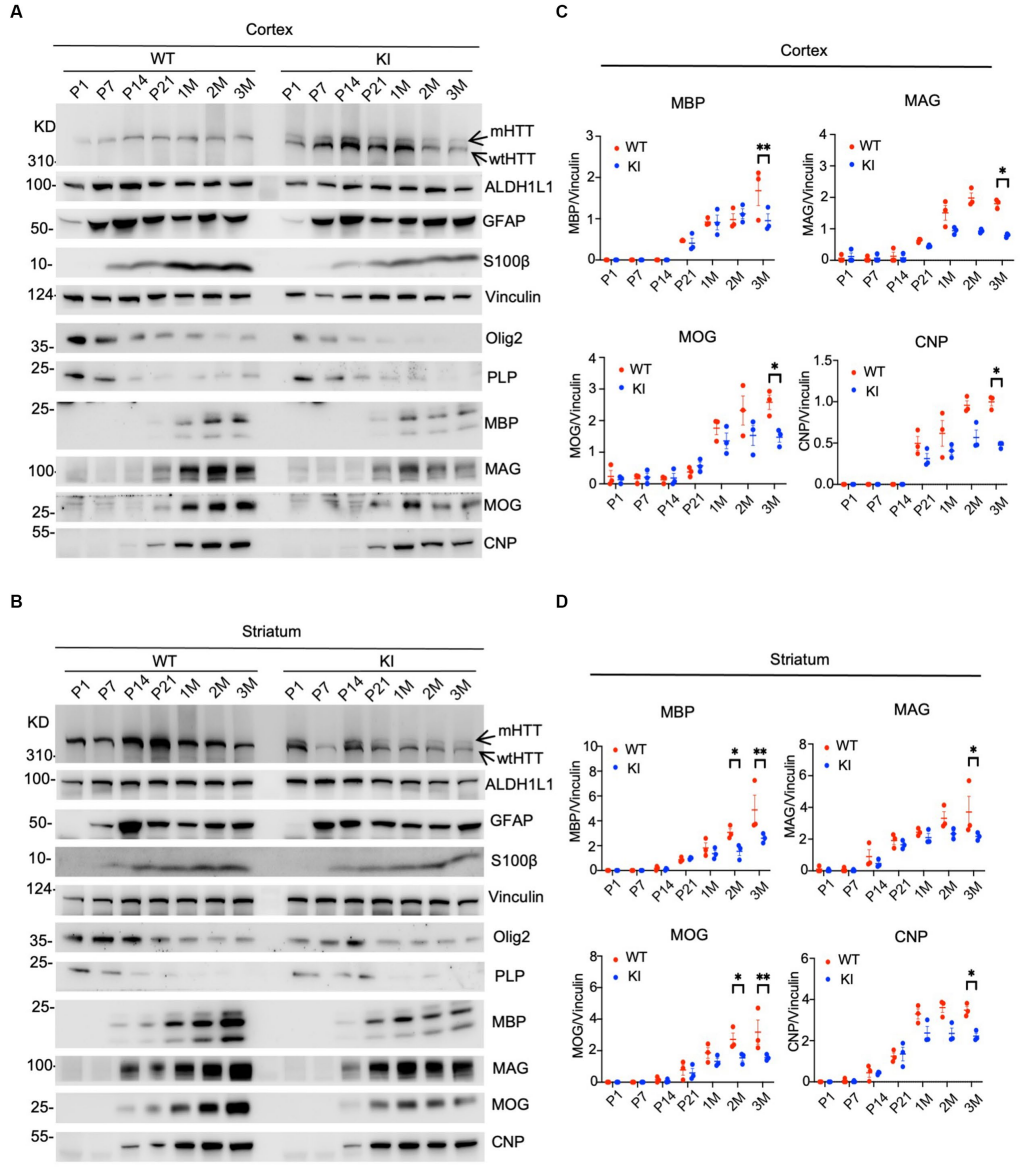
Selective reduction in myelin proteins in HD KI mouse brain. Western blotting analysis of the prefrontal cortex **(A)** and striatum **(B)** of WT and HD KI mice at the ages of P1, P7, P21, 1 M, 2 M, 3 M. The blots were probed with antibodies to the astrocytic proteins ALDH1L1, GFAP, S100β and oligodendrocytic proteins olig2, PLP, MBP, MOG, MAG, CNP, Vinculin served as an internal control. Quantification of western blots of the prefrontal cortex **(C)** and striatum **(D)**, normalized to vinculin. P, postnatal; M, month; WT, wild type; KI, knock-in. The data were presented as mean+/-SE (*n* = 3 independent experiments from 3 mice per genotype). ^*^p < 0.05, ^**^p < 0.01.

We performed western blotting to examine the expression levels of glial proteins. This assay can validate the expression of mutant HTT and also quantitatively measure the relative levels of glial proteins by comparing with the loading control protein on the same blots. In the cortex, heterozygous HD KI mice expressed wild type and mutant HTT that showed less immunoreactivity to the anti-HTT than WT HTT (Figure 1A, arrows). The expression of both WT and mutant HTT appeared to be quite stable from P1 to 3 months. Astrocytic proteins GFAP and S100beta were markedly increased from P1 to P7 and then stably expressed from P7 to 3 months, whereas ALDH1L1, which labels glial cells at different states, was more consistently expressed at different postnatal days (Figure 1A). However, oligodendrocytic proteins (olig2 and PLP) were more abundant at P1-P7 and declined from P7, suggesting that the expression of oligodendrocytic proteins is dynamic at the postnatal stage. However, mutant HTT does not seem to affect the expression of these glial proteins in the cortex when compared with that of WT mice. In contrast, myelin proteins (MBP, MAG, MOG, and CNP) were reduced in their expression when compared with WT mice. This conclusion is also supported by quantification of the ratios of glial proteins to the loading control vinculin on western blots, and a significant reduction of these myelin proteins was found at 3 months of age (Figurse 1A,C).

Similar changes were also seen in the striatum of HD KI mice, where glial proteins were unchanged between WT and KI mice, but myelin proteins (MBP, MAG, MOG, and CNP) showed reduced levels, especially at 3 months of age (Figures 1B,D). Thus, examining postnatal mice at multiple time points revealed that myelin proteins were selectively reduced in HD KI mice.

### 3.2. Decreased myelin protein staining in HD KI mice

**Figure 2:**
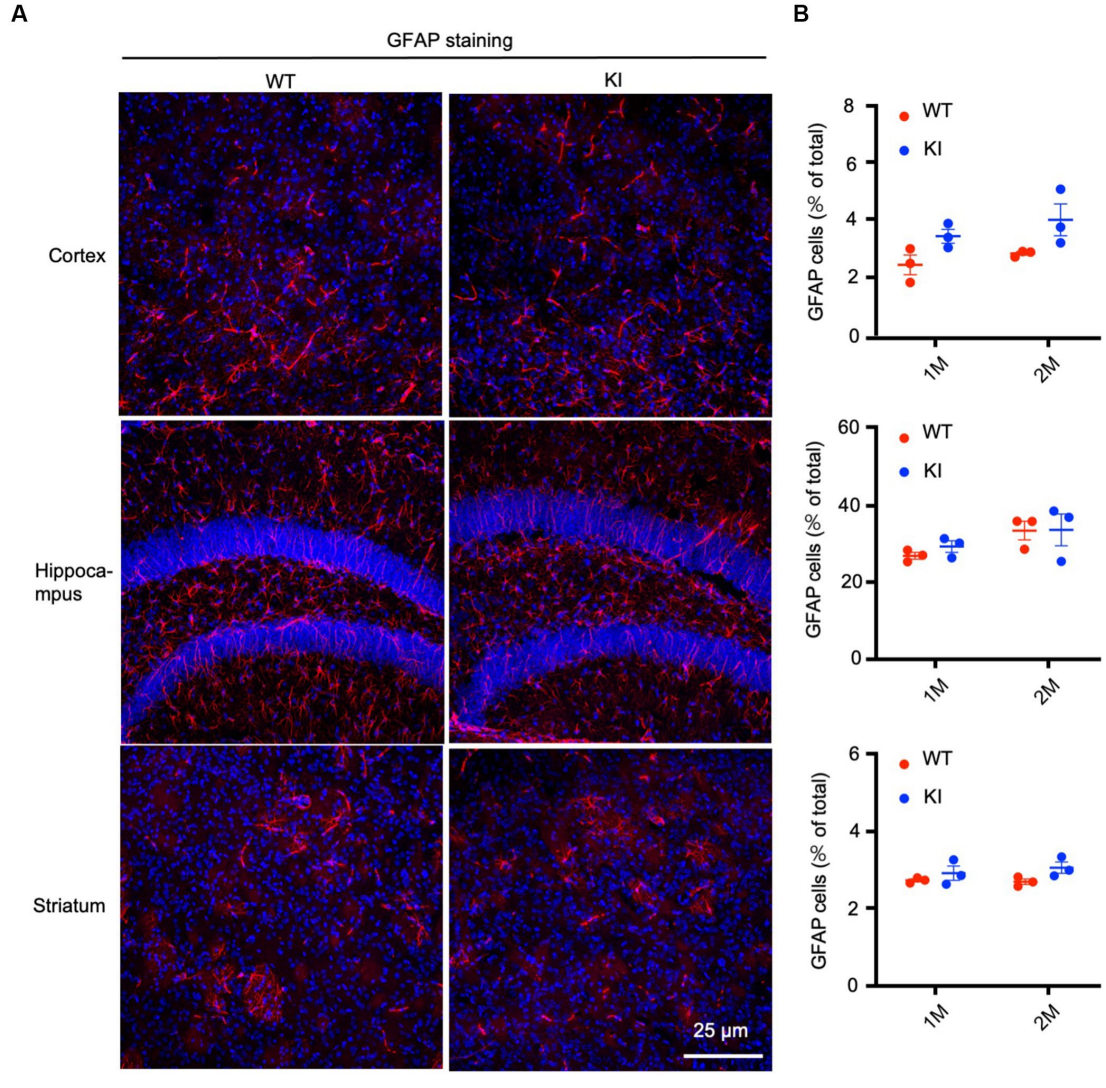
No significant alteration in astrocyte numbers in postnatal HD KI mice. **(A)** Immunofluorescence staining of the prefrontal cortex, striatum and hippocampus for the astrocytic protein GFAP in 2-month-old mice. **(B)** Quantification of GFAP-positive cell rate (% of total cells). The data were presented as mean+/-SE (*n* = 3 independent experiments from 3 mice per genotype). P, postnatal; M, month. WT, wild type; KI, Knock-in.

To confirm the Western blotting results, we performed immunocytochemistry using antibodies specific to the astrocytic protein GFAP and oligodendrocytic protein Oligo2. We used heterozygous HD KI mice and wild type littermates of the same age and isolated their brains at different days after birth, ranging from P1 to 3 months. Immunostaining of different brain regions, including the cortex, hippocampus, and striatum, of WT and HD KI mice at 1 and 2 months after birth did not reveal any significant differences in GFAP labeling (Figure 2A). Counting the numbers of GFAP-positive cells (*n* = 3 mice per group) also showed no significant difference between WT and HD KI mice (Figure 2B). These results suggest that mutant HTT does not affect the development of astrocytes, as there were no significant differences in GFAP-positive cells between HD KI and WT mice.

**Figure 3:**
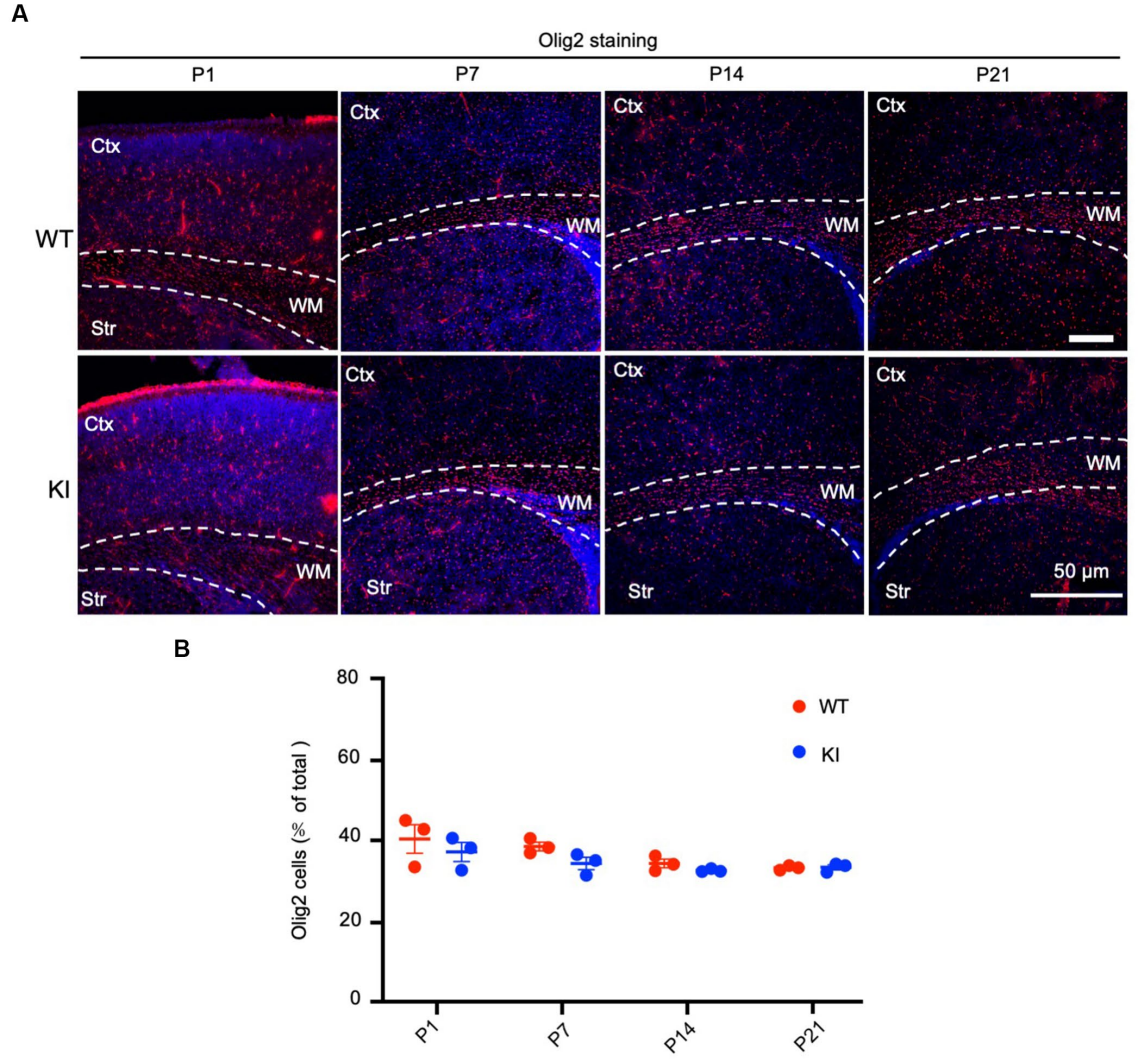
No significant alteration in oligodendrocyte numbers in early postnatal HD KI mice. **(A)** Immunofluorescence staining of the mouse brain for the oligodendrocytes with an antibody to olig2. WT and HD KI mice at the ages of P1, P7, P14 and P21 were examined. The brain region contains the cortex (Ctx), whithe matter (WM) in the corpus callosum, and striatum (Str). **(B)** Quantification of olig2-positive cell rate (% of total cells). The data were presented as mean+/-SE (*n* = 3 independent experiments from 3 mice per genotype). P, postnatal; WT, wild type; KI, knock-in.

To assess the number of oligodendrocytes, we used an antibody to oligo2, a protein specifically expressed in oligodendrocytes, to immunolabel WT and HD KI mice at P1, P7, P14, and P21. The examination did not reveal any obvious changes in oligo2 labeling in the brain regions containing the cortex, corpus callosum, and striatum when compared with WT mice (Figure 3A). Quantification of the oligo2-positive cells (*n* = 3 animals per group) showed similar numbers of these cells (Figure 3B). Thus, mutant HTT does not alter the numbers of oligodendrocytes during early brain development.

**Figure 4:**
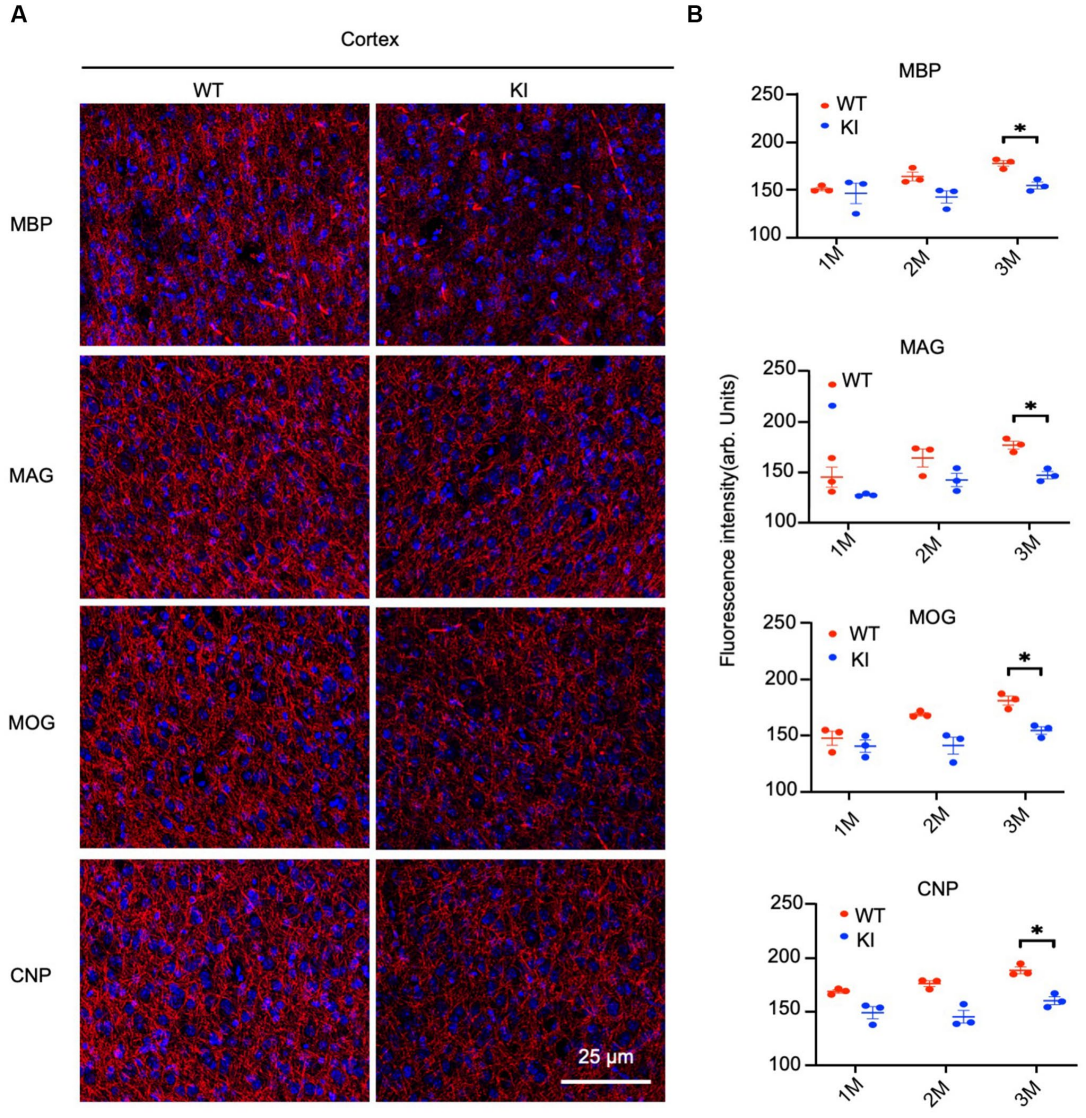
Reduced myelin protein staining in the cortex in HD KI mouse brain. **(A)** Immunofluorescence staining of the prefrontal cortex for myelin proteins MBP, MOG, MAG and CNP. **(B)** Quantification of myelin proteins fluorescence density. The data were presented as mean+/-SE (n = 3 independent experiments from 3 mice per genotype). P, postnatal; M, month. ^*^p < 0.05, WT, wild type; KI, knock-in.

**Figure 5:**
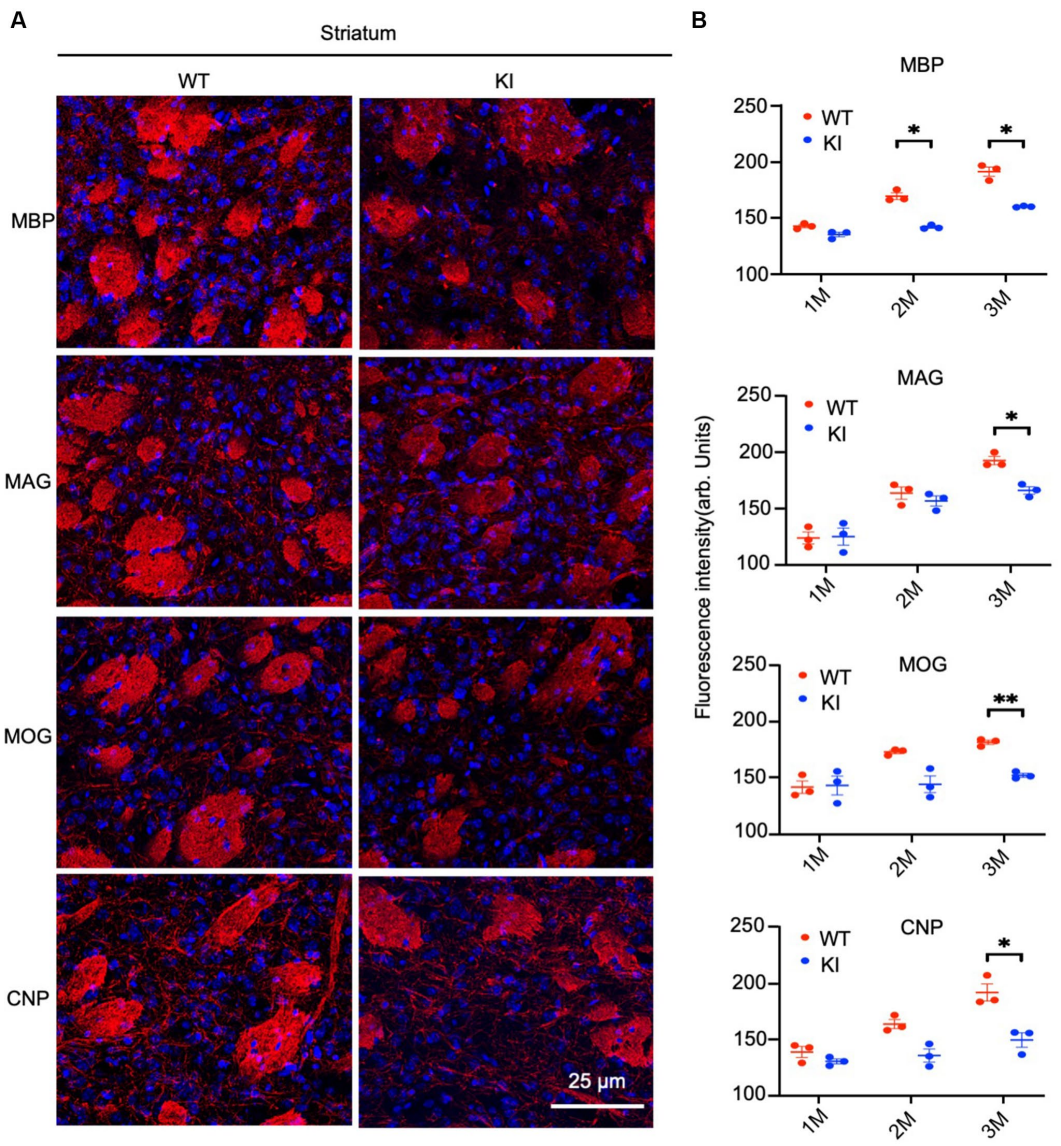
Reduced myelin protein staining in the striatum in HD KI mouse brain. **(A)** Immunofluorescence staining of striatum for myelin proteins MBP, MOG, MAG and CNP. **(B)** Quantification of myelin proteins fluorescence density. The data were presented as mean+/-SE (*n* = 3 independent experiments from 3 mice per genotype). P, postnatal; M, month. ^*^p < 0.05, WT, wild type; KI, knock-in.

By staining the cortex with antibodies to myelin proteins (MBP, MAG, MOG, and CNP), we observed decreases in these proteins in HD mice at 3 months of age (Figure 4A). Quantification of immunofluorescent staining intensity also verified the decreases in the cortex of HD KI mice compared to WT mice (Figure 4B). Similarly, immunostaining of MBP, MAG, MOG, and CNP in the striatum was also reduced in HD KI mice at 3 months of age (Figures 5A,B). These results are consistent with the western blotting results in Figure 1 and demonstrate that mutant HTT can reduce myelin protein expression when mice reach 3 months of age.

### 3.3. Decreased myelination in HD KI mice

**Figure 6:**
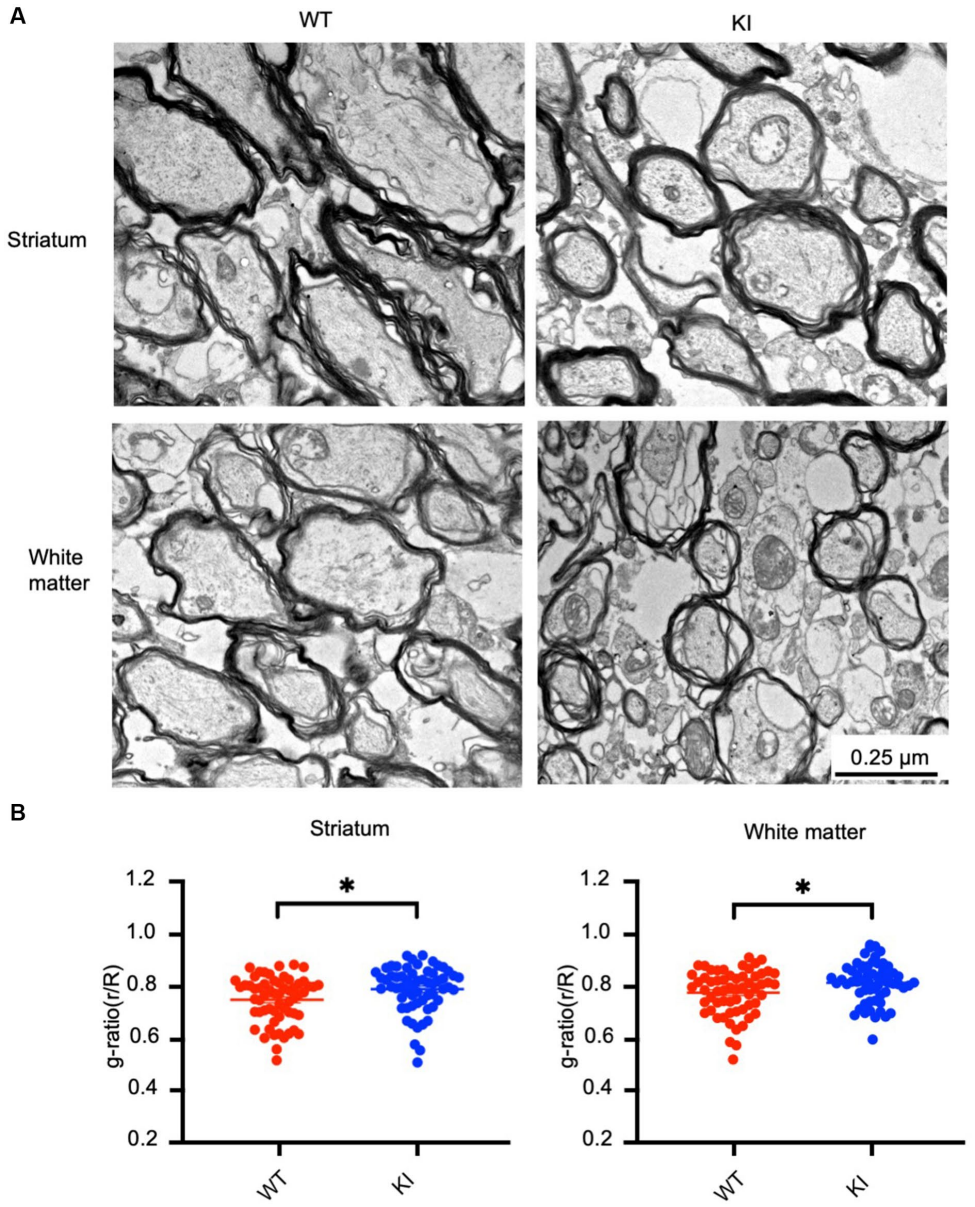
Reduced myelination in the HD KI mouse brain at 3 months of age. **(A)** Electron microscopic graphs of the striatum and subcortical white matter. Scale bar: 0.25 μm. **(B)** G ratios (r/R) of axons in WT and HD KI mice. WT: wild type, KI: knock-in. The data were presented as mean+/-SE and obtained from counting 60 axons per group. ^*^p < 0.05.

Next, we aimed to investigate whether mutant Htt causes any myelination defects in HD KI mice at 3 months of age. Electron microscopy revealed myelinated axons in the striatum and white matter (WM) of both WT and HD KI mice. However, the thickness of myelin in HD KI mice appeared to be thinner than that of WT mice (Figure 6A). To confirm this, we performed a quantitative analysis of g-ratios (the inner axonal diameter to the total outer diameter) (Figure 6B). The ratio was increased in the striatum and white matter in HD KI mice compared to WT mice, reflecting thinner myelin in HD KI mice (Figure 6B). We did not observe any obvious evidence of axon degeneration in HD KI mice, suggesting that reduced myelination is an early pathological change prior to obvious degeneration in HD.

### 3.4. RNAseq analysis of postnatal HD mice

**Figure 7:**
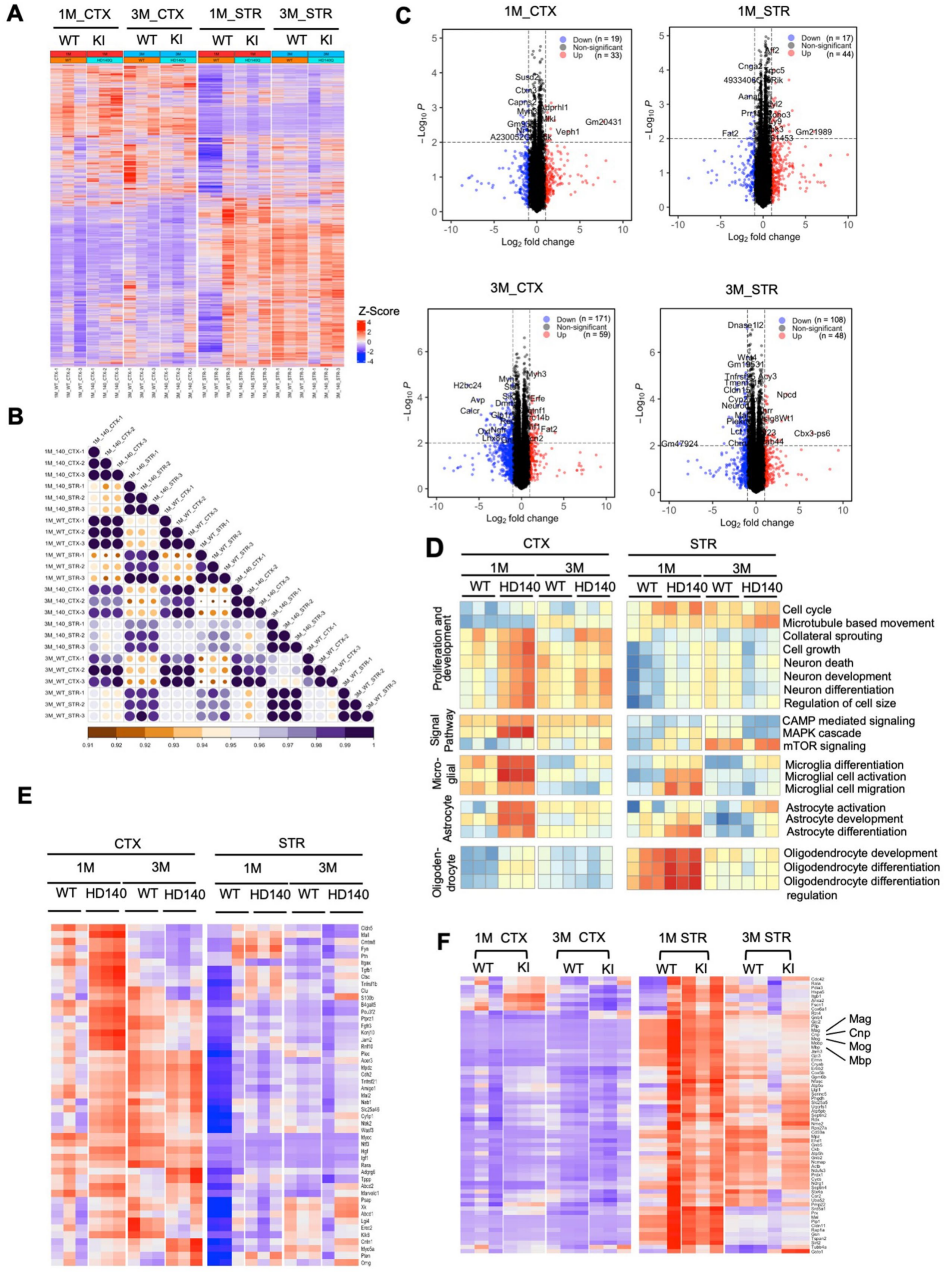
Postnatal wild-type (WT) and Huntington’s disease knock-in (HD KI) mice show similar gene expression patterns in the cortex and striatum. **(A)** The heatmap displays gene expression in WT and HD KI mice (n = 3 per group), with red indicating higher expression and blue indicating lower expression. **(B)** The correlation between each sample is plotted through a heatmap. The scale bar indicates correlation coefficients ranging from 0.9 to 1.0. **(C)** The volcano plots of differentially expressed genes (DEGs) (adjusted *p*-value <0.01, |fold change| > 1.0) between HD KI and WT at 1 and 3 months. **(D)** Pathway analysis results suggest minimal differences between HD KI and WT in five neuron and glial cell-related pathway sets, with red indicating pathway activation and blue indicating pathway inhibition. **(E)** The expression matrix of myelination-related genes in the cortex and striatum tissues of postnatal WT and HD KI mice is shown. **(F)** Comparing gene expression of a subset of genes for myelin sheath (*n* = 3 mice) showing slight alterations in HD KI mice. CTX, cortex; STR, striatum, 1 M, 1-month-old; 3 M, 3-month-old.

Transcriptional dysregulation is a significant molecular change in HD (Malla et al., 2021), which is observed in HD KI mice and correlates with the age-dependent nuclear accumulation of mutant HTT (Langfelder et al., 2016). HD KI mice do not exhibit significant alterations in gene expression until 6 months of age (Langfelder et al., 2016), consistent with the apparent nuclear accumulation of N-terminal mutant HTT at this age (Yang et al., 2020). We conducted RNAseq analysis to investigate whether postnatal HD KI mice (*n* = 3) display any altered gene expression. Analysis of HD KI mice at 1 and 3 months did not reveal any significant changes in global gene expression in the cortex and striatum compared to WT mice (*n* = 3) (Figures 7A,B). The volcano plots of differentially expressed genes (DEGs) also showed minimal numbers of altered genes (19–33 at 1 month of age and 171–59 at 3 months of age for cortex, 17–44 at 1 month of age and 108–48 at 3 months of age for striatum), although there is a trend towards increased numbers with aging (Figure 7C). We also used quantitative RT-PCR to compare the expression of several genes involved in the regulation of neuronal and glial differentiation and maturation, but did not identify any that displayed obvious alterations in HD KI mice at 3 months of age (Supplementary Figure S1). Characterization of genes for neuronal and glial cell differentiation and development supports the idea that mutant HD mice do not exhibit a deficiency in the numbers of glial cells at the postnatal stage (Figure 7D). Analyzing the expression of genes for myelination did not reveal significant changes in HD KI mice either (Figure 7E; Supplementary Figure S1A). Although their average values revealed alterations in some subset genes for myelin sheath, the expression of MBP, MAG, MOG, and CNP appeared to be slightly up-regulated in HD KI mice when compared with those of WT mice (Figure 7F) which is consistent with quantative PCR resulte (Supplementary Figure S1). The lack of reduction of these myelin genes in postnatal HD KI mice is in line with the absence of obvious nuclear accumulation of mutant HTT.

## 4. Discussion

The discovery of the effects of mutant HTT on early neuronal development raises an important idea that late-onset neurodegeneration in HD may be initiated by early defects in neuronal development (Cepeda et al., 2019; van der Plas et al., 2020; Humbert and Barnat, 2022). Since mutant HTT is also expressed in glial cells and glial dysfunction contributes to HD neuropathology (Shin et al., 2005; Bradford et al., 2009, 2010; Khakh and Sofroniew, 2014; Huang et al., 2015; Diaz-Castro et al., 2019; Lee et al., 2022), it is interesting to investigate whether mutant HTT affects glial development, thereby contributing to early neuronal development defects. In the current study, we found that mutant HTT does not affect glial development, as there are no obvious alterations in the numbers of astrocytes and oligodendrocytes in the brains of postnatal HD mice. These findings suggest that neuronal toxicity of mutant HTT occurs much earlier than glial toxicity, whereas non-autonomous effects resulting from mutant HTT in glial cells may be important for facilitating HTT toxicity and disease progression.

Although previous studies have shown that mutant HTT can affect both astrocytes and oligodendrocytes (Shin et al., 2005; Bradford et al., 2009, 2010; Khakh and Sofroniew, 2014; Huang et al., 2015; Diaz-Castro et al., 2019; Lee et al., 2022; Sun et al., 2022), it has never been investigated which types of glial cells are preferentially affected at the early disease stage. Addressing this issue is important for understanding the development and progression of HD, as astrocytes and oligodendrocytes play distinct roles in maintaining neuronal function. Astrocytes can regulate synaptic neurotransmitters and release growth factors to maintain neuronal function, whereas oligodendrocytes are critical for myelination, which is essential for axonal conductivity and function (Haydon, 2001; Allen and Lyons, 2018; Duncan et al., 2021; Liu et al., 2023). We found that mutant HTT reduced the expression level of myelin proteins but not oligodendrocytic numbers, suggesting that mutant HTT affects the oligodendrocytic function that produces myelin proteins.

The findings that mutant HTT reduces myelin protein expression and myelination are consistent with several previous reports that mutant HTT can affect axonal integrity and myelination (Shirendeb et al., 2012; Marangoni et al., 2014; Huang et al., 2015; Teo et al., 2016; Rosas et al., 2018; Bourbon-Teles et al., 2019; Ferrari Bardile et al., 2019). These previous studies investigated the effects of mutant HTT in transgenic mouse models. However, it remains unknown how full-length mutant HTT at the endogenous level affects glial cells, particularly at the early stages of the disease. Our early study demonstrated that selective expression of transgenic N-terminal mutant HTT in oligodendrocytes can induce much more severe demyelination and axonal degeneration in PLP-150Q mice (Huang et al., 2015; Yin et al., 2020), whereas other HD transgenic mice displayed different extents of demyelination phenotypes (Teo et al., 2016; Ferrari Bardile et al., 2019, 2021). Thus, the level of toxic HTT products is critical for dysfunction of oligodendrocytes.

In the current study, we examined HD KI mice in which full-length mutant HTT is expressed at the endogenous level. Although the extent to which mutant HTT affects myelination is milder in HD KI mice than in transgenic mouse models of HD, the current finding points to the fact that demyelination is an early pathological event in HD when full-length mutant HTT is expressed under physiological conditions. It is known that there is an age-dependent accumulation of N-terminal mutant HTT in the nucleus, which affects gene expression, whereas full-length mutant HTT is predominantly distributed in the cytoplasm (Zhao et al., 2016). In line with this, HD KI mice expressing 111-175Q do not show obvious alterations in gene expression at 2 months of age until 6 months of age (Langfelder et al., 2016). Our RNAseq data indicate that the mutant HTT does not significantly affect gene expression in the early stages of the disease. For instance, at 1 month, only 17 genes were found to be down-regulated and 44 genes were upregulated in the striatum of HD KI mice. At 3 months, the number of altered genes increased to 108 down-regulated and 48 upregulated genes. When compared to the altered gene expression in the striatum of adult HD KI mice, which could be more than 2000 genes up-or down-regulated (Langfelder et al., 2016), the changes found in our HD KI mice at the early disease stage are minor. These changes reflect the age-dependent nuclear effects of mutant HTT, which is consistent with the fact that nuclear accumulation of mutant HTT depends on the accumulation of N-terminal HTT fragments in the nucleus. However, the minor nuclear effects of mutant HTT seen in our RNAseq analysis do not account for the decreased myelin proteins at 3 months, as both RNA-seq and RT-PCR did not reveal decreased levels of myelin protein mRNAs. Thus, the reduced myelin proteins are likely due to the cytoplasmic effects of mutant HTT rather than nuclear mutant HTT that can affect gene expression.

The production of myelin proteins seems to be regulated by complicated mechanisms. For example, MBP mRNA needs to be transported from the nucleus to the plasma membrane to be translated locally at the axon-glial contact site (Müller et al., 2013). This transport relies on kinesin-mediated intracellular trafficking (Lyons et al., 2009) and intracellular signaling regulation (Laursen et al., 2011). In addition, axon-glial signaling is also critical for myelination, and axonal diameter or electrical activity influences myelination (Krämer-Albers and White, 2011; Müller et al., 2013). Since cytoplasmic mutant HTT can affect a variety of cellular functions, including intracellular trafficking, mRNA translation or stability, and various signaling pathways (Bates et al., 2015; Saudou and Humbert, 2016; Fu et al., 2019; Eshraghi et al., 2021), the defective myelination in postnatal HD KI mice highlights the effect of cytoplasmic mutant HTT on myelination in the early stages of HD. These findings offer additional insight into the pathogenesis of HD and have therapeutic implications for halting or preventing HD neuropathology.

## Data availability statement

The datasets presented in this study can be found in online repositories. The names of the repository/repositories and accession number(s) can be found at: https://www.ncbi.nlm.nih.gov/bioproject/PRJNA982440/.

## Ethics statement

The animal study was reviewed and approved by the Institutional Animal Care and Use Committees at Jinan University.

## Author contributions

XG and X-JL conceived the study. SY and JM conducted major experiments. YL, MP, Haz, HoZ, and JL participated in some experiments. LC analyzed RNAseq data. SL and DH provided advice. SY, XG, and X-JL wrote the manuscript with contributions and approval of submitted version by all authors.

## Funding

This study was supported by National Natural Science Foundation of China (81830032, 82071421, and 82271902) and Natural Science Foundation of Guangdong Province (2021A1515012526).

## Conflict of interest

The authors declare that the research was conducted in the absence of any commercial or financial relationships that could be construed as a potential conflict of interest.

## Publisher’s note

All claims expressed in this article are solely those of the authors and do not necessarily represent those of their affiliated organizations, or those of the publisher, the editors and the reviewers. Any product that may be evaluated in this article, or claim that may be made by its manufacturer, is not guaranteed or endorsed by the publisher.
