# Supplementary figures and images for: Mutant HTT does not affect glial development but impairs myelination in the early disease stage
Source: Front Neurosci. 2023 Jul 19;17:1238306. doi: 10.3389/fnins.2023.1238306 (PMC10394243; doi:10.3389/fnins.2023.1238306)

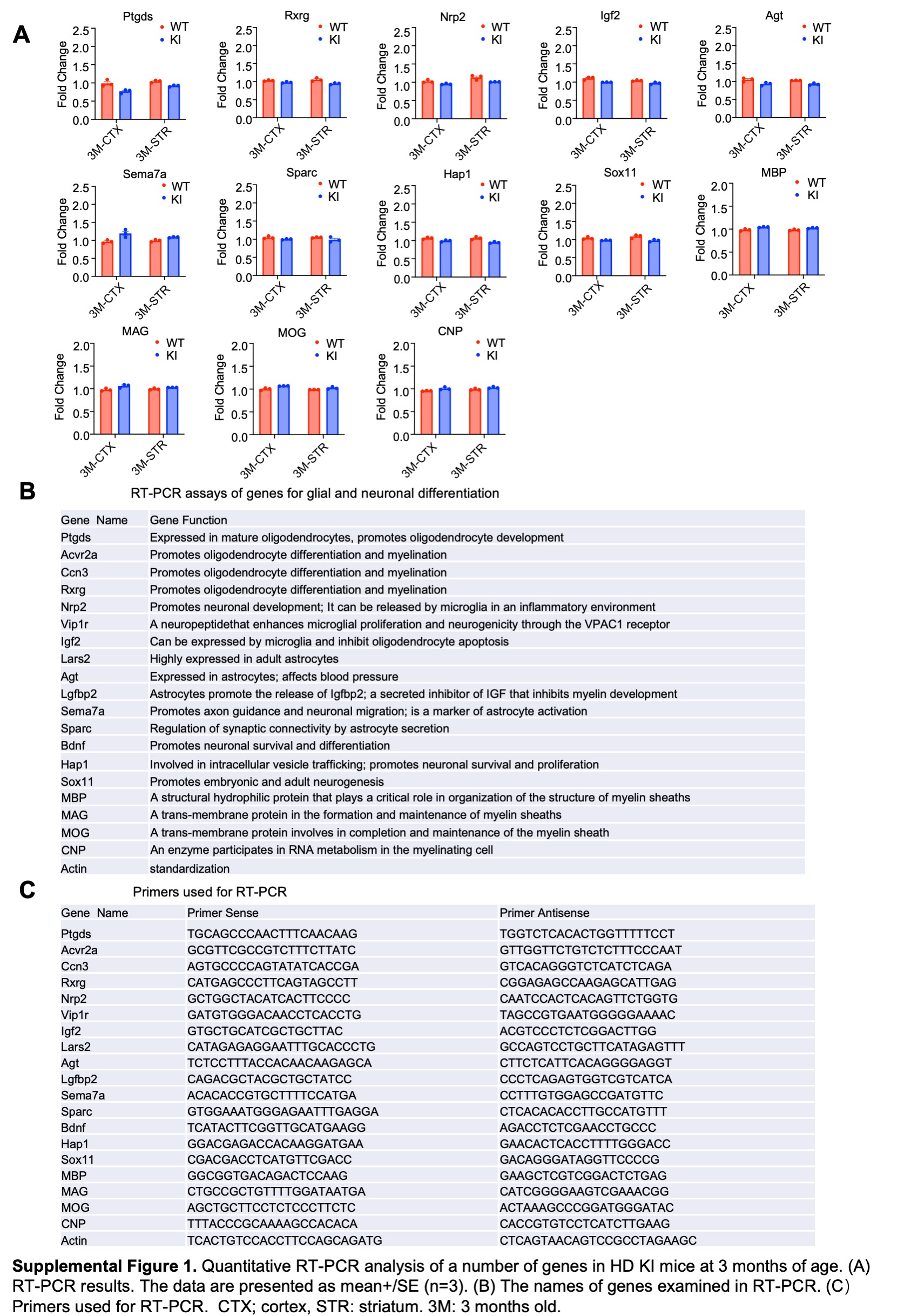

Supplement: Supplementary file 1 [file Image_1.jpeg]
